# The Negative Affect Hypothesis of Noise Sensitivity

**DOI:** 10.3390/ijerph120505284

**Published:** 2015-05-18

**Authors:** Daniel Shepherd, Marja Heinonen-Guzejev, Kauko Heikkilä, Kim N. Dirks, Michael J. Hautus, David Welch, David McBride

**Affiliations:** 1 School of Public Health, Auckland University of Technology, Auckland 0627, New Zealand; E-Mail: daniel.shepherd@aut.ac.nz; 2 Department of Public Health, University of Helsinki, PO Box 41, Helsinki FI-00014, Finland; E-Mails: marja.heinonen@helsinki.fi (M.H.-G.); kauko.heikkila@helsinki.fi (K.H.); 3 School of Population Health, University of Auckland, Auckland 1142, New Zealand; E-Mails: k.dirks@auckland.ac.nz (K.N.D.); d.welch@auckland.ac.nz (D.W.); 4 School of Psychology, University of Auckland, Auckland 1142, New Zealand; E-Mail: m.hautus@auckland.ac.nz; 5 School of Medicine, University of Otago, Dunedin 9054, New Zealand; E-Mail: david.mcbride@otago.ac.nz

**Keywords:** noise sensitivity, negative affect, annoyance

## Abstract

Some studies indicate that noise sensitivity is explained by negative affect, a dispositional tendency to negatively evaluate situations and the self. Individuals high in such traits may report a greater sensitivity to other sensory stimuli, such as smell, bright light and pain. However, research investigating the relationship between noise sensitivity and sensitivity to stimuli associated with other sensory modalities has not always supported the notion of a common underlying trait, such as negative affect, driving them. Additionally, other explanations of noise sensitivity based on cognitive processes have existed in the clinical literature for over 50 years. Here, we report on secondary analyses of pre-existing laboratory (*n* = 74) and epidemiological (*n* = 1005) data focusing on the relationship between noise sensitivity to and annoyance with a variety of olfactory-related stimuli. In the first study a correlational design examined the relationships between noise sensitivity, noise annoyance, and perceptual ratings of 16 odors. The second study sought differences between mean noise and air pollution annoyance scores across noise sensitivity categories. Results from both analyses failed to support the notion that, by itself, negative affectivity explains sensitivity to noise.

## 1. Introduction

Noise sensitivity describes a vulnerability to the negative health impacts associated with environmental noise exposure. Noise sensitive individuals are more likely to attend to sound and evaluate it negatively (e.g., perceive it as threatening or annoying), have stronger emotional reactions to sound and, consequently, have greater difficulty habituating to sounds [1]. Job [2] conceptualized noise sensitivity as internal states (be they physiological or psychological) that typically amplify arousal to noise. Weinstein [3] postulated that noise sensitivity could be understood as a general tendency to express negative judgments of a person’s immediate environment, and noise sensitive individuals should therefore be sensitive to other environmental stimuli such as odor, brightness or temperature. However, as an identifiable reaction modifier to noise, noise sensitivity is currently well described but as yet not sufficiently explained. This lack of etiological progress is likely caused by the fact that noise sensitivity mechanisms may be multifactorial in nature, with factors influencing the degree of noise sensitivity acting either independently or interactively.

Given the high prevalence of noise sensitivity in both general [4] and clinical [5] populations, it is of interest to further investigate its underlying mechanisms. Noise sensitivity, by definition, manifests negative evaluations of noise. The challenge is to determine the cause of these negative evaluations. Explanations of noise sensitivity currently retrievable from the literature can be grossly classified into one of three approaches. Firstly, and the most common explanation found in the *epidemiological* literature [6,7], is that noise sensitivity reflects negative affectivity, an over-willingness to complain about objects and events beyond one’s control. Secondly, and the most common explanation found in the *clinical* literature, are explanations based on bottom-up cognitive processes by which noise-induced memory and attentional deficits lead to annoyance or distress. These cognitive explanations rely heavily upon information-processing models of auditory distractors [8]. Noise-induced interference of cognitive processes has been well described elsewhere [9,10]. The third approach, in which noise sensitivity is attributed to hypervigilance to noise sources due to fear and anxiety [1,11,12], is only rarely encountered, and usually used in relation to a specific noise source (e.g., aviation noise). However, the boundaries between these three approaches are not always clear cut, for example, some argue that noise-related negative affectivity maybe preceded by a previously developed attribution of threat from the noise [1]. 

The focus of this paper is the Negative Affectivity hypothesis of noise sensitivity. Negative affectivity (NA) was formally defined by Watson and Clark [13], with high levels describing a stable and pervasive personality dimension characterized by individuals more inclined to report distress, discomfort, and dissatisfaction, even in the absence of obvious stressors. In the noise literature the concept of NA first emerged as “critical” tendencies of noise sensitive individuals to negatively evaluate a broad range of environmental stimuli, and NA is often (erroneously) represented by the trait measure of neuroticism. Broadbent [14] and Weinstein [3] both noted that those who reported high levels of noise annoyance or noise sensitivity had a greater propensity to negative evaluations. In a sample of female psychiatric patients, Stansfeld *et al.*, [15] likewise reported a positive relationship between noise sensitivity and both neuroticism and reactivity across a selection of environment stimuli, but cautions against attributed noise sensitivity exclusively to a “the tendency of neurotic people to complain about their environment.” (p. 251). In subsequent studies Stansfeld and colleagues concluded that noise sensitivity is related to NA even in the absence of psychiatric morbidity [1], though note that care must be taken to distinguish NA from a general sensitivity to environmental quality, whereby noise sensitivity is associated with a greater vulnerability to the noxious effects of noise [16]. Other studies have shown only weak relations between noise and neuroticism [17], and between noise sensitivity and neuroticism [18].

The NA hypothesis states that noise annoyance is not especially stimulus-oriented, rather that sound only has to be audible to be annoying. Thus, sound level or other qualities such as modulation would not always be useful predictors of noise annoyance. Studies have supported this notion, indicating that noise sensitivity is independent of noise exposure [12]. Additionally, the NA approach would predict annoyance responses to be uniform across sensations, irrespective of the sensory modality of origin. Such a proposition is also relevant to the Environmental Intolerance approach [19], which argues that noise sensitivity is part of a more global sensitivity to environmental stimuli, albeit with a focus on neurophysiological rather than personality explanations [20]. To test the veracity of the NA hypothesis, one could examine the covariance between noise annoyance and, for example, olfactory-related annoyance. Conveniently, the relationship between noise and olfactory sensitivity, including chemical intolerance, has already been explored in the literature [21].

Nordin *et al.* [22] assessed differences in odor and noise intolerance between persons with electromagnetic hypersensitivity and healthy controls, reporting that the former scored significantly higher than the controls on all chemical sensitivity and noise sensitivity scales. Palmquist *et al.* [23] found significant relationships between intolerance to odorous/pungent chemicals and sounds, for both self-reported and diagnosed intolerance. However, other studies have differentiated noise sensitivity and chemical intolerance as unique entities [24]. While one study suggested that noise sensitive individuals could be more annoyed by tobacco smoke [25], another suggested that those with impaired sensory gating self-medicate with tobacco in order to mitigate noise sensitivity [26]. Investigations into the association between noise sensitivity and annoyance (as measured by “discomfort thresholds”) to a variety of stimuli including noise, light, and temperature extremes (hot/cold) only uncovered significant relationships between noise sensitivity and noise discomfort [27]. More generally, inconclusive findings were reported by Reynolds *et al.* [28] who investigated whether neuroticism, of which negative affect is a component, undermines cognitive performance (e.g., arithmetic and IQ tasks) performed in the presence of acoustic distracters. Their results could be taken to provide only partial support for the NA hypothesis. Finally, epidemiological studies reporting significant correlations between NA and noise sensitivity are typically small, with the magnitude of the variance explained at a level of around 5% [7]. Thus, while NA might be a statistically significant predictor of noise sensitivity, it fails to account for the majority of the variance, indicating that other factors need to be considered.

The current study aims to further investigate noise sensitivity by way of secondary analyses of two pre-existing datasets. These datasets afford an examination of the relationship between noise sensitivity and response to olfactory stimuli, which could potentially lend support to the NA hypothesis. Specifically, if the NA hypothesis holds, then strong associations between noise sensitivity and evaluations of olfactory stimuli would be expected.

### Study One

As with sound, an individual’s hedonic response to odor is highly subjective [29]. Whether an odor is liked (or disliked) is typically the most immediate response, even if it cannot be identified. Study One entails a secondary analysis of odor perception, noise sensitivity, and noise annoyance data originally collected as part of a study describing links between odor perception and electrophysiological indices. The NA hypothesis would predict a significant positive correlation between noise sensitivity and noise annoyance, and a negative correlation between noise sensitivity and odor pleasantness. We consider that odor annoyance is conceptually identical to odor pleasantness, both manifesting hedonic judgments and thus effectively synonyms. In an as yet unpublished study, the first author recently collected sound perception data and noted a correlation between pleasantness and annoyance ratings of *r* = −0.83, indicative of redundancy. Furthermore, the magnitudes of these two correlations may be expected to be approximately equal. It would further be expected that NA would amplify the relationship between noise sensitivity and odor intensity, as it would be expected that a general tendency to complain would be accompanied by exaggerations of exposed quantities. The same may hold for odor familiarity, whereby NA would be expected to exaggerate the frequency of exposures.

## 2. Material and Methods

### 2.1. Participants

Seventy-four participants aged 20–69 years (*M_age_* = 33.51, *SD* = 12.28) were recruited, totaling 31 males and 43 females. Participants consisted of staff and students from the University of Auckland and the Auckland University of Technology, New Zealand, recruited via institutional email advertisements. The methods and procedures used in this study were reviewed and endorsed by the University of Auckland Human Participants Ethics Committee prior to commencement of the study.

### 2.2. Noise Sensitivity

Noise sensitivity was estimated using the Noise Sensitivity Questionnaire (NOISEQ) scale [30] which measures global noise sensitivity as well as noise sensitivity for different domains of everyday life: Leisure, work, sleep, communication, and habitation. There are 35 NOISEQ items, each requiring the respondent to indicate their degree of agreement to statements about their responses to noise using a five-point Likert-type scale, modified from the original four-point NOISEQ scales [30]. Global noise sensitivity is computed as the average of the leisure, work, habitation, communication and sleep subscales, with data recoded so higher means indicate greater levels of noise sensitivity. For this dataset, the Cronbach’s alpha was an acceptable 0.855. 

### 2.3. Noise Annoyance

Six questions regarding annoyance to different noise sources were combined to create a total noise annoyance score. The six items measured general annoyance to neighborhood noise, and annoyance to noise while reading, watching television, relaxing, working, or sleeping. Each item was rated on a three-point category scale: 0 = “No”/1 = “Sometimes”/2 = “Yes”. Ratings to the six items were summed to produce a Total Score, with higher scores indicating higher annoyance. The Cronbach’s Alpha for the six items was an acceptable 0.724.

### 2.4. Odor Perception

**Table 1:** Mean pleasantness, intensity, and familiarity ratings for 16 odor types and associated Total Scores. Bivariate and partial (in parentheses and controlling for age) correlation coefficients between olfactory ratings and noise sensitivity are also presented.

	Pleasantness		Intensity		Familiarity	
Odorant	Mean	*r*/*r*_age_	Mean	*r*/*r*_age_	Mean	*r*/*r*_age_
Orange	4.08	−0.134 (−0.187)	3.86	0.038 (0.088)	4.34	−0.103 (−0.051)
Leather	2.72	−0.04 (−0.196)	3.11	0.008 (0.058)	2.84	−0.354 * (−0.390 *)
Cinnamon	3.84	−0.082 (−0.152)	3.78	0.068 (0.130)	3.97	0.144 (0.169)
Peppermint	4.25	−0.087 (−0.088)	4.58	0.001 (0.061)	4.84	−0.035 (0.071)
Banana	3.63	0.028 (−0.030)	4.01	0.170 (0.17)	4.32	−0.109 (−0.091)
Lemon	4.05	−0.04 (−0.020)	3.68	−0.005 (0.039)	4.18	0.012 (0.059)
Liquorice	3.57	0.125 (0.074)	3.85	−0.068 (−0.100)	4.31	−0.118 (−0.151)
Turpentine	2.23	−0.249 * (−0.314 *)	3.47	0.031 (0.095)	2.27	−0.123 (−0.145)
Garlic	3.03	−0.076 (−0.103)	4.60	0.161 (0.212)	4.72	0.027 (0.036)
Coffee	3.41	0.252 * (0.280 *)	3.62	0.197 (0.237 *)	3.89	0.099 (0.141)
Apple	4.05	0.026 (0.047)	3.36	−0.051 (−0.077)	3.23	0.171 (0.148)
Cloves	3.03	0.034 (−0.041)	3.84	0.044 (0.051)	3.49	0.050 (−0.002)
Pineapple	4.20	0.029 (0.29)	3.45	0.049 (0.074)	3.77	0.093 (0.129)
Rose	4.00	0.096 (0.46)	3.79	0.212 (0.184)	3.79	−0.015 (−0.015)
Anise	3.59	0.111 (0.026)	3.55	0.228 (0.136)	3.80	0.088 (−0.058)
Fish	1.53	0.106 (0.097)	4.77	0.052 (0.062)	4.64	−0.088 (−0.080)
**Total Score**	54.878	0.035 (−0.065)	60.329	0.133 (0.164)	62.135	−0.039 (−0.041)

* *p* < 0.05.

Responses to odors involved the identification of the odor and ratings of odor pleasantness, intensity, and familiarity. A commercially-available product, the Sniffin’ Sticks Olfactory Test, was utilized [31], consisting of 16 distinct odors (Table 1). The Sniffin’ Sticks test employs pen-like odor-dispensing devices, each housing a tampon containing either a liquid odorant or odorant dissolved in propylene glycol to a total volume of 4 ml. The pens each have tight-fitting lids designed to eliminate odor contamination. When a lid is removed, the pen dispenses a constant concentration of odor [31]. Following Distel *et al.* [32], participants were required to verbally rate the odor’s familiarity, pleasantness, and intensity, on a five-point scale. On these scales, “1” represented the most familiar, pleasant, and intense rating and “5” the least. This part of the assessment is not part of the original Sniffin’ Sticks battery, but was added to assess the properties of these odor characteristics and how they might be related to measures such as personality.

### 2.5. Procedure

The experimental session began with the administration of the Sniffin’ Sticks odor identification test. During this task, participants were also asked to rate the familiarity, pleasantness, and intensity of each odor. Following removal of the cap, the pen was positioned approximately two centimeters under the participant’s nostrils for three seconds. There was at least a 30-s delay prior to the presentation of the next odor to prevent carryover effects. No feedback was given to the participant during the task. Following the presentation and rating of odors, the participants were given a battery of surveys to complete, including noise sensitivity and noise annoyance questionnaires.

### 2.6. Analysis

Preliminary analysis was conducting using zero-order and first-order correlation coefficients. All tests were two-tailed and participant age was controlled for as age is a known covariate of noise sensitivity [11]. Cohen’s effect size categories are useful when assessing the strength of correlation coefficients: Small (*r* < 0.3), moderate (*r* = 0.3 to 0.5), and large (*r* > 0.5). Associations between noise sensitivity and noise annoyance, and noise sensitivity and odor perception were explored using hierarchical multiple linear regression, ensuring the effects of participant age were partialled out.

## 3. Results and Discussion

As previously reported [11], age was positively correlated with noise sensitivity (*r* = 0.331, *p* = 0.004), necessitating its inclusion as a covariate. In relation to gender, independent samples *t*-tests uncovered no significant differences between males and females in terms of noise sensitivity, percentage correct odor identification, and the Total Scores for pleasantness, intensity, or familiarity ratings (*p* > 0.05). Consequently, gender was not used as a between-group factor in subsequent analyses.

### 3.1. Associations between Noise Sensitivity and Odor Perception

The mean ratings for pleasantness, intensity, and familiarity are presented in Table 1. Across the entire sample, peppermint and pineapple were judged the most pleasant, and fish the least. Garlic, peppermint and fish were deemed the most intense, as well as the most familiar. Nonparametric Mann Whitney tests uncovered no significant differences in noise sensitivity between those able to identify specific odors and those who couldn’t (*p* > 0.05). The correlation between noise sensitivity and overall percentage correct identification was likewise non-significant (*r* = −0.058, *p* = 0.311). This null result is of interest, as certain psychopathologies (e.g., schizophrenia) are characterized by both odor identification deficits and noise sensitivity [33]. As such, it is possible that this finding would not be replicated in clinical populations.

To further explore the associations between odor perception and noise sensitivity, both zero-order and first-order (controlling for age) correlation coefficients were computed (Table 1). Significant correlations between noise sensitivity and the pleasantness of both turpentine (a negative coefficient) and coffee (a positive coefficient) were found, both increasing in magnitude when controlling for age. However, if Bonferonni *post hoc* corrections are applied, statistical significance ceases for these two relationships. While the turpentine finding is consistent with the NA hypothesis, it should be noted that the overall variance accounted for is less than 10%, and thus other factors need to be accounted for. Additionally, the correlation between noise sensitivity and the pleasantness Total Score was small (*r* = 0.035), even after controlling for age (*r*_age_ = −0.065), and, pertinently, is non-significant. The positive correlation between rated pleasantness of coffee odor and noise sensitivity would not be predicted by the NA hypothesis.

In relation to intensity, only a single significant positive correlation was noted (once age had been controlled for), between noise sensitivity and the perceived intensity of coffee. If the NA hypothesis holds, it might be expected that intensity ratings would be exaggerated by noise-sensitive individuals. While the correlation between noise sensitivity and the Total Score for intensity is of greater magnitude than that for pleasantness, it is still non-significant, with noise sensitivity explaining less than 3% of the variability in the total intensity score. Similarly, the NA hypothesis would predict that noise-sensitive individuals would overestimate their exposure to specific odors, and this would be reflected in the familiarity ratings. Intriguingly, a moderate negative correlation between noise sensitivity and leather was uncovered; one could speculate that this finding is consistent with the notion of Idiopathic Environmental Intolerance (IEI) if noise sensitive individuals avoid leather due to its tactile properties. In relation to the total familiarity score, however, there seems little evidence to support over-reporting of exposure due to NA.

### 3.2. Associations between Noise Sensitivity, Noise Annoyance and Odor Perception

Multiple linear regression analyses were undertaken to test whether, after controlling for age, there would be a significant association between noise sensitivity and noise annoyance. Such a finding would merely replicate previous reports in the literature [7], though would endow a degree of validity upon this aspect of the design. The result would likewise be predicted by the NA hypothesis. Inspection of Table 2 confirms the significant association between noise sensitivity and noise annoyance. Further, the NA hypothesis would predict that a significant association between noise sensitivity and odor perception would also emerge. Table 2 reveals no statistically significant relationship between noise sensitivity and odor pleasantness (β = −0.066, *p* > 0.05), a finding that impeaches the veracity of the NA hypothesis. Non-significant findings were also found with odor intensity and odor familiarity.

To conclude, the results do not offer definitive support for the NA hypothesis. As a secondary analysis, there are naturally limitations, for example, the use of the ‘pleasantness-unpleasantness’ descriptors with the odor data, *versus* ‘not annoying-annoying’ with the noise annoyance data. Additionally, in relation to the small number of significant relationships found in Table 1, further research is required to determine whether there exists selective responding to certain stimuli (e.g., coffee and turpentine), or if in fact the data simply manifest a random pattern of Type I errors.Scrutiny of Table 1 reveals that the bulk of the odors are food related, and that two-of-the-three significant correlations (Turpentine and Leather) are non-food related. Thus future research could consider expanding the range of odors.

**Table 2:** Four Hierarchical multiple linear regressions between noise sensitivity (always the dependent variable) and (a) noise annoyance, (b) odor pleasantness, (c) odor intensity, and (d) odor familiarity. To control for participant age, it was always included in the first step.

Variables	*R*^2^	Δ*R*^2^	*F*	*B*	beta	*t*
Step 1: Age	0.110	0.110 *	8.883 *	0.013	0.331	2.980 *
Step 2: Age	0.273	0.163 **	13.315 **	0.010	0.273	2.664 *
(a) Noise Annoyance				0.081	0.408	3.988 **
Step 1: Age	0.110	0.110 *	8.883 *	0.013	0.331	2.980 *
Step 2: Age	0.114	0.004	4.556 *	0.013	0.345	3.002 *
(b) Odor Pleasantness				−0.004	−0.066	−0.576
Step 1: Age	0.110	0.110 *	8.883 *	0.013	0.331	2.980 *
Step 2: Age	0.135	0.024	5.443 *	0.014	0.362	3.199 *
(c) Odor Intensity				0.006	0.159	1.401
Step 1: Age	0.110	0.110 *	8.883 *	0.013	0.331	2.980 *
Step 2: Age	0.112	0.002	4.466 *	0.013	0.328	2.924 *
(d) Odor Familiarity				−0.003	−0.044	−0.392

* *p* < 0.05, ** *p* < 0.001.

### 3.3. Study Two

Study Two involved three datasets and a selection of common variables. These variables, including measures of noise sensitivity, and both noise and air quality annoyance, afford tests of hypotheses generated from the NA approach. Firstly, if noise sensitivity reflects an underlying trait of negative appraisal, then average noise-induced annoyance ratings would be expected to be invariant between highly noise sensitive individuals residing in areas differing in noise exposure. Secondly, the qualitative form of the relationship between noise annoyance and noise sensitivity would not depend on noise exposure levels. Thirdly, similarly, there would be significant differences in the mean air quality ratings across levels of noise sensitivity, irrespective of exposure, again indicating that negative disposition may be influencing annoyance ratings. The broader approach used in Study Two in some respects mirrors that described by Smith and Stansfeld [34]. 

## 4. Material and Methods

### 4.1. Participants

Data for Study Two were obtained in New Zealand’s two largest cities: Auckland (2010; 2013) and Wellington (2012). The Auckland data consists of two datasets. The first set compromised the “Motorway” (*n* = 373) and the “Non-Motorway” (*n* = 253) samples. The Motorway sample consists of residents living within 50 meters of Auckland’s motorway system, with noise levels estimated to be approximately 76 dB(A) LDN [35]. The Non-Motorway reference sample contains residents from two areas within the Auckland region, located at least 2 kilometers away from any significant source of environmental noise (e.g., industry or roads), and with noise levels estimated to be around 55 dB(A) LDN. The second Auckland dataset consists of two samples from the central business district (CBD), denoted “CBD-Traffic” (*n* = 134) and “CBD-Pedestrian” (*n* = 65); the latter was from a road closed to traffic, while the former was the main thoroughfare through the central city [36]. Mobile monitoring of noise levels was undertaken using a commercially-available dosimeter (CEL-350 dBadge, Casella) providing sound level measurements (dB LA_eq_) every minute. Each location was monitored for 10 h, with dB LA_eq_ values of 70.98 (CBD-Traffic) and 69.55 (CBD-Pedestrian) being recorded. The Wellington data comprises the “Airport” sample (*n* = 87) and the “Non-Airport” sample (*n* = 93). The Airport sample resided within 250 meters of the Wellington International Airport’s runway, with aviation noise levels estimated at 62 dB(A) LDN, with peak values legislated to stay below 75 dB(A) L_max_ [37]. The Non-Airport reference sample consisted of residents living on the city’s urban/suburban border, and was far from the airport’s main flight path. The Motorway and Non-Motorway samples were socioeconomically matched (middle to high deprivation) and were from suburban neighborhoods, whilst the Airport and Non-Airport samples were also matched (low to middle deprivation), but were neighborhoods on the suburban/urban boundary. Rudimentary demographic profiles for the six samples are presented in Table 3.

**Table 3:** Demographic profiles of the six samples. The values are raw frequencies with percentages presented in brackets. Differences in proportions between samples within a dataset are tested using Pearson’s chi-square tests. Note that percentages may be affected by missing data.

	Motorway	Non-Motorway	Airport	Non-Airport	CBD-Traffic	CBD-Pedestrian
**Sex**						
**Male**	93 (34.6)	105 (43)	28 (32.6)	31 (33.3)	76 (56.7)	36 (55.4)
**Female**	171 (63.6)	140 (57)	58 (67.4)	61 (65.6)	57 (42.5)	29 (44.6)
**Chi-Square**	(χ^2^(2) = 3.29, *p* = 0.078)		(χ^2^(1) = 0.05, *p* = 0.824)		(χ^2^(1) = 0.055, *p* = 0.815)	
**Age Group (Years)**						
**18-20**	7 (2.6)	4 (1.6)	3 (3.4)	2 (2.2)	27 (20.1)	20 (38.8)
**21-30**	36 (13.4)	14 (5.5)	7 (8)	8 (8.6)	58 (43.3)	19 (29.2)
**31-40**	47 (17.5)	68 (26.9)	16 (18.4)	18 (19.4)	20 (14.9)	7 (10.8)
**41-50**	55 (20.4)	56 (22.1)	16 (18.4)	20 (21.5)	6 (4.5)	7 (10.8)
**51-60**	47 (17.5)	40 (15.8)	14 (16.1)	20 (21.5)	13 (9.7)	6 (9.2)
**61-70**	35 (13)	43 (17.0)	16 (18.4)	16 (17.2)	4 (3)	5 (7.7)
**70+**	37 (13.8)	23 (9.1)	14 (16.1)	8 (8.6)	5 (3.7)	1 (1.5)
**Chi-Square**	(χ^2^(7) = 18.51, *p* = 0.005)		(χ^2^(7) = 4.527, *p* = 0.75)		(χ^2^(2) = 10.357, *p* = 0.11)	
**Noise Sensitivity**						
**Low**	98 (38)	91 (34.9)	40 (46)	39 (41.9)	24 (17.9)	20 (30.8)
**Moderate**	125 (50)	139 (53.3)	33 (37.9)	41 (44.1)	73 (54.4)	25 (38.5)
**High**	26 (10.4)	31 (11.9)	14 (16.1)	13 (14)	36 (26.9)	20 (30.8)
**Chi-Square**	(χ^2^(2) = 1.159, *p* = 0.56)		(χ^2^(2) =7.15, *p* = 0.699)		(χ^2^(2) = 5.357, *p* = 0.069)	

### 4.2. Survey

Data for the Motorway, Non-Motorway, Airport, and Non-Airport, samples were taken from a larger survey entitled “Wellbeing and Neighborhood Survey” [35,38]. Items pertinent to the current analysis were the seven items probing annoyance to environmental factors, including air pollution (“air pollution from traffic”, “air pollution from household chimneys”, “other, please specify”) and noise (“noise from traffic”, “noise from other neighbors”, “other noise, please specify”), where “traffic” was unspecified and source non-specific. These items were presented by way of a five-point scale ranging from “not annoyed at all” to “extremely annoyed”. Noise sensitivity was measured using a single item made up of three response categories representing low, moderate, and high noise sensitivities. The final section of the survey sought personal information including gender and age. Each house received two copies of the questionnaire, delivered in their post-box, a participant information sheet, and stamped-addressed envelopes. Though not identical, the survey used to gather data from the CBD-Pedestrian and CBD-Traffic samples contained a number of items found in the aforementioned Wellbeing and Neighborhood Survey, including the noise sensitivity and annoyance questions. In this study, surveys were distributed to pedestrians in transit and were returned immediately upon completion. No information regarding residency was recorded. These studies were approved by the Auckland University of Technology Ethics Committee (08/256).

### 4.3. Statistical Analysis

Data analyses were carried out using SPSS Version 18. Differences in mean annoyance scores across noise sensitivity categories were tested using analyses of variance and Bonferonni *post hoc* tests, while differences across areas, but within a noise sensitivity band, were tested using independent samples *t*-tests. Covariates (*i.e.*, age) were included if significant associations were uncovered between factors during preliminary analyses.

## 5. Results and Discussion

Table 3 shows the frequency distribution of noise sensitivity in each of the six samples, with proportions of high noise sensitivity ranging from 10 to 16 percent across the four suburban-type samples (all but the final two columns). This pattern suggests that highly noise sensitive individuals are not less likely to live in close proximity to a motorway or an airport, and therefore the choice of house location may not be influenced by the individual’s self-rated noise sensitivity, as might be expected. In the two urban samples (CBD-Traffic and CBD-Pedestrian), the prevalence rates are higher, possibly due to high noise exposures eliciting negative responses in individuals who might not otherwise have thought of themselves as sensitive to noise. Previous European studies have estimated the prevalence of high noise sensitivity in urban residents to be between 40 to 50 percent [39,40] higher than those obtained in the current study. This discrepancy may in part be explained by the fact that the current study did not distinguish between residents and non-residents, unlike the aforementioned European studies, and so factors such as sense-of-place may not have influenced ratings.

### 5.1. Noise Annoyance Ratings

**Figure 1:**
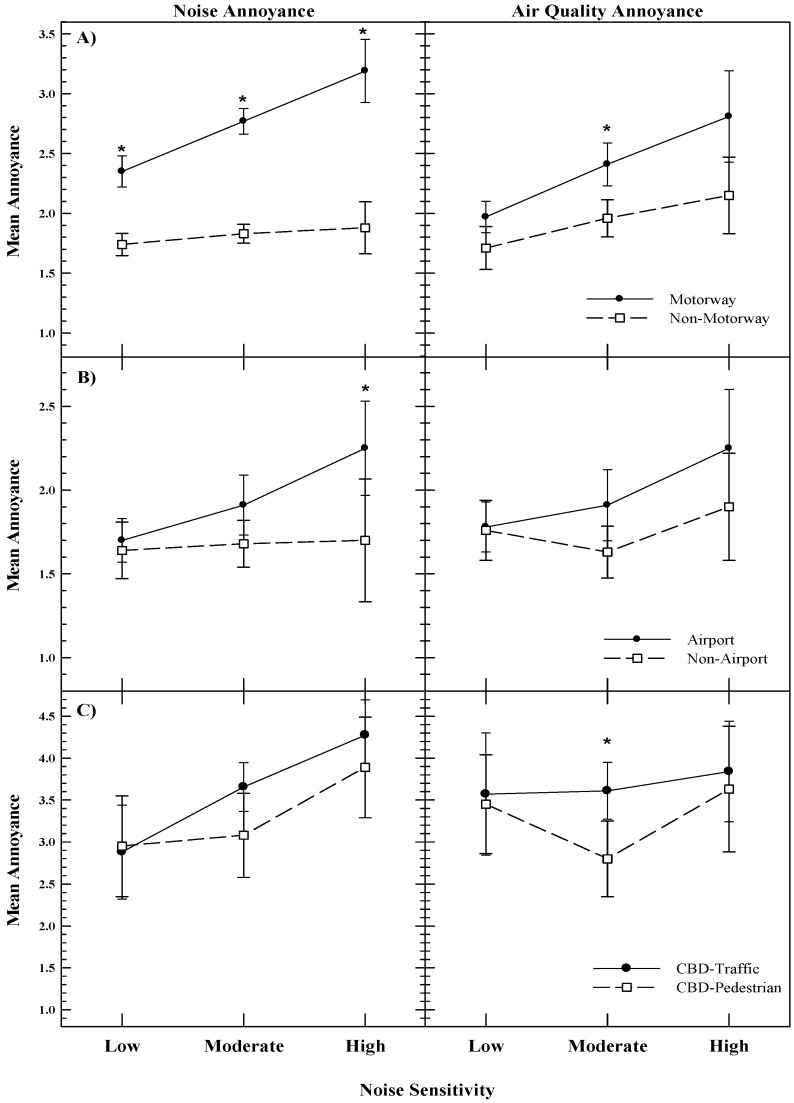
Mean annoyance to noise and air pollution as a function of self-reported noise sensitivity for samples collected in two New Zealand cities: Auckland (top and bottom panels) and Wellington (middle panel). Whiskers are 95% confidence intervals, with asterisks indicating significant differences between means (*p* < 0.05). Note the different scales on the *y*-axes.

The mean noise annoyance ratings are displayed in Figure 1 (left column). The expected positive relationship between noise sensitivity and noise annoyance is evident for all but two samples: The Non-Motorway and Non-Airport samples. Heinonen-Guzejev *et al.* [41] compared self-reported measures of noise annoyance and noise sensitivity against information on noise maps in the Metropolitan Area of Helsinki, Finland. They reported that transportation-induced annoyance was higher among individuals with higher noise sensitivity. That the high noise sensitivity groups in both the Non-Motorway and Non-Airport samples exhibited equivalent noise annoyance ratings to non-sensitive individuals might be taken as support for the restoration hypothesis, whereby quiet areas provide relief for sensitive individuals [42].

The NA hypothesis suggesting that mean noise annoyance ratings would be equivalent across groups of high noise sensitive individuals, irrespective of noise exposure, was tested for two scenarios. Firstly, two tests compared groups of highly noise sensitive individuals exposed to different levels (and sources) of noise (Motorway *vs.* Non-Motorway and Airport *vs.* Non-Airport) in which non-significant differences would support the NA hypothesis. Secondly, a test was carried out comparing groups of high sensitive individuals exposed to similar noise levels (CBD-Traffic *versus* CBD-Pedestrian) in which a significant difference in annoyance ratings would suggest responses reflecting processes unrelated to noise exposure. The left column in Figure 1 shows the mean noise annoyance ratings for the three datasets, with asterisks indicating significant differences between groups (e.g., Motorway *versus* Non-Motorway) within each of the three levels of noise sensitivity. For the high noise sensitivity groups, significant results are obtained when comparing Motorway *versus* Non-Motorway and Airport *versus* Non-Airport samples, and a non-significant result when comparing the CBD-Traffic *versus* CBD-Pedestrian samples. 

These findings do not provide support for the NA hypothesis, but might be taken by some (e.g., Smith & Stansfeld [34]) to support the so-called “noise vulnerability hypothesis”, which asserts that noise has its greatest impact on vulnerable (*i.e*., noise sensitive) individuals. Here the interaction between noise exposure and noise sensitivity was significant for both motorway (*F*(2,501) = 4.278, *p* = 0.014), and airport (*F*(2,178) = 4.499, *p* = 0.035) data. Smith and Stansfeld [34], using measures of “everyday errors” obtained from a small sample exposed to either low or high levels of aircraft noise, failed to uncover significant interactions and thus did not support for the noise vulnerability hypothesis. That the two sets of results between Smith and Stansfeld [34] and the current study do not align may be explained by a number of factors, including the former study’s lack of power, or due to the differences in the variables (*i.e*., every day errors vs. annoyance). Furthermore, the interaction between noise exposure and noise sensitivity found in this study supports the notion that the effect of noise sensitivity upon the noise exposure—noise annoyance relationship is not only additive in nature [12], as demonstrated by the flat functions associated with low noise sensitivity and step functions with high sensitivity. 

Furthermore, if noise sensitivity is uncoupled from acoustic factors such as level and thus reflects other processes (e.g., negative affect), an invariant relationship between noise annoyance and noise sensitivity should be observed, irrespective of the noise exposure context. Figure 1 shows that the noise context does shape the association between noise annoyance and noise sensitivity. For samples exposed to greater noise levels (*i.e.*, the Motorway and Airport groups), a proportional relationship between noise annoyance and sensitivity is demonstrated, with significant differences in mean noise annoyance scores between levels of noise sensitivity for both the Motorway (*F*(2,505) = 5.888, *p* = 0.003) and Airport (*F*(2,85) = 4.908, *p* = 0.010) groups. Subsequent *post hoc* tests revealed significant differences in mean noise annoyance ratings between the least and moderate sensitivity groups (*p* = 0.004), and the least and highly sensitive group (*p* < 0.001) in the motorway locale, and the low and high noise sensitivity groups (*p* = 0.007) in the Airport sample. However, for samples exposed to relatively lower noise levels (*i.e.*, Non-Motorway and Non-Airport), significance across the three noise sensitivity groups was not obtained (*p* > 0.05). For samples exposed to equivalent noise levels (*i.e.*, CBD-Traffic and CBD-Pedestrian), a main effect of noise sensitivity was noted for the CBD-Traffic data (*F*(2,129) = 9.083, *p* < 0.001: all *post hoc* tests *p* < 0.05), though the CBD-Pedestrian data were marginal (*F*(2,62) = 2.847, *p* = 0.050), with *post hoc* tests revealing a difference between the least and highly sensitivity groups (*p* = 0.044). Thus, analysis of noise annoyance across noise sensitivity levels likewise fails to definitively support the NA hypothesis.

### 5.2. Air quality Annoyance Ratings

The NA hypothesis would predict that, for any given sample, mean air quality annoyance ratings will differ significantly when comparing two groups differing in noise sensitivity, with the higher sensitivity group having higher annoyance. Only one of six of the air quality annoyance functions displayed in Figure 1 (second column) differed significantly across noise sensitivity groups: The Motorway sample (*F*(2,505) = 6.403, *p* = 0.002). Significant differences in mean air pollution annoyance ratings between the least and moderate noise sensitivity groups (*p* = 0.024), and the least and the highly sensitive group (*p* = 0.003), were noted.

Further scrutiny of the plotted air quality data invites comparisons between areas within a single level of noise sensitivity, though interpretation of the trends become more complex. If noise sensitivity simply reflects an underlying trait of NA, then for the highly noise sensitive groups, air quality annoyance ratings should not differ significantly between the different areas, irrespective of air pollution exposure. For the high noise sensitivity samples, the NA hypothesis is supported by the three datasets of interest. However, while this finding *prima facia* supports the NA hypothesis, consideration of the data set at large and alternative explanations must be accommodated. Specifically, it is noted across the three datasets that, for the low noise sensitivity groups, there are no significant differences between areas. Furthermore, with the exception of the motorway/non-motorway data, there are no statistical differences between the low and high noise sensitive groups in terms of mean air quality annoyance (*p* > 0.05). Thus, *ipso facto*, a null result between the two areas at a single level of noise sensitivity cannot be taken as evidence of NA. Interestingly, for both the motorway/non-motorway and CBD-Traffic/CBD-Pedestrian data sets, there is statistical significance for the moderate noise sensitivity samples, though in the current context it is difficult to derive meaning from these significant results other than perhaps representing a Type I error.

In summary, the results show that only one of the three hypotheses supports the NA hypothesis; specifically, there is a mean difference in air pollution annoyance across the three noise sensitivity groups for the Motorway sample, with larger means associated with larger degrees of noise sensitivity. Even so, there is more than one explanation for this difference, which somewhat attenuates the support for personality factors as influencing responses to environmental pollutants. Pertinently, those who report greater sensitivity to noise may do so because, given their living environment, they are more exposed to noise and more aware of their sensibilities. Consequently, they may in fact be more exposed to greater levels of air pollution. For example, Persson *et al.* [7] report significant correlations between noise sensitivity and self-reported annoyance to traffic exhaust fumes, as well as between noise annoyance and physical measures of nitrogen oxide exposure. Of further note is that while a significant difference in noise annoyance means was found between the motorway and non-motorway areas at the high noise sensitivity level, no such finding emerged from the air quality data. This finding is in opposition to that expected if individuals have a general sensitivity to environmental quality [1]. 

A limitation of the study is that when rating annoyance to air quality, the participants may be basing their ratings all, or in part, on the visual aspects of air pollution. However, the impact of visual pollution will in all likelihood be minimal, as New Zealand’s vehicle fleet is relatively modern, and cities are coastal and exposed to sea breezes. Indeed, visual air pollution is rare and usually reported in the national media if it occurs. Furthermore, while we cannot discount annoyance due to visual factors, this would merely displace the influence from one modality (olfaction) to another (visual), and the same hypotheses would hold. Note, however, that in all three noise annoyance plots (Figure 1, left column), the mean annoyance scores in the moderately noise sensitive group lie almost halfway between the mean scores for the least noise sensitive group and the highly sensitive group. This suggests that the noise sensitivity scale is appropriate and robust, despite being only a single item scale and the validity of such scales being doubted in previous research [43].

## 6. General Discussion

Our study consisted of secondary analyses of multiple pre-existing datasets, each affording scrutiny of the NA hypothesis of noise sensitivity. Findings derived from comparing olfactory perceptual scales and noise sensitivity metrics (Study One) offered little support for the NA hypothesis, while the results obtained from several epidemiological datasets (Study Two) likewise failed to definitively uphold the predictions of the NA hypothesis. Thus, it can be argued here that two very different approaches to data collection (laboratory-based *versus* epidemiological-based methods) produced similar findings. 

Our findings fail to conform to contemporary opinion [44] or selected data reported in the literature [22]. Sensitivity to sensory dimensions such as brightness, color, pain, smell, and touch, have correlated significantly with noise sensitivity measures in previous studies [15,22]. The divergence between our findings and those of Nordin *et al.* [22] may be explained by the separation of their sample into EMF-sensitive and EMF-non-sensitive groups, with the former group possibly exhibiting traits consistent with negative affectivity. If it is accepted that noise sensitivity itself is multifactorial, as the current authors would argue, then it would be perilous to interpret the finding of Nordin *et al.* [22] as proof that noise sensitivity by its nature manifests general environmental sensitivities, or that noise sensitivity is sufficiently explained by negative affectivity. It is more difficult to reconcile our results with Stansfeld *et al.* [15] as they recruited only females, and utilized a composite scale of ‘general sensitivity’ questions that included a single smell item (“I am very aware of smells and scents”) which does not necessarily reflect negative evaluations, and was, unfortunately, not analyzed in isolation.

Alternatively, information processing approaches, originating from cognitive psychology, would predict that while a significant positive association between noise sensitivity and noise annoyance should be observed, no association between noise sensitivity and olfactory-based judgments would be expected. Specifically, this approach would predict that annoyance may differ between the sensory modalities due to different neural infrastructures and sensory processing characteristics. For example, while the visual, auditory, gustatory and tactile modalities all utilize parts of the thalamus as a relay station, the olfactory modality takes a very different pathway through the olfactory bulb and olfactory (Pyriform) cortex [45]. Hence an uncoupling between noise sensitivity and olfactory evaluations might be expected on the basis of differing underlying neurological processes. In Study One, the significant correlation between noise sensitivity and coffee would not be predicted by the NA hypothesis, though potentially could be accounted for by information processing models. For example, impaired sensory gating, by which the thalamus cannot filter out irrelevant stimuli, is common in schizophrenia [46]. Moreover, sensory gating can be augmented using nicotine, with schizophrenics commonly self-medicating with this substance [26]. Some studies indicate that caffeine improves transmission in the peripheral and central brain auditory pathways [47], and future studies looking at the effects of caffeine upon auditory processes between noise sensitive and noise tolerant individuals would be of interest.

Noise sensitivity may be subsumed by the Environmental Intolerance approach, which is characterized by the attribution of several, multisystem symptoms (e.g., headaches) to specific environmental exposures such as odorous/pungent chemicals, everyday sounds, and electromagnetic fields (EMF) [27]. It has been hypothesized that individual differences in limbic system reactivities and central nervous system sensitizabilities underlie vulnerabilities to environmental stimuli [20]. Individuals who are sensitive to both chemicals and noise might be among the most vulnerable to limbic dysfunction and to the sensitization of the limbic and central nervous systems as a result of multiple environmental factors [21]. The amygdala, one of several brain regions that modulates startle reactions to unexpected noise [48], is also the most dominant regions of the brain when responding to chemical stimuli [49]. However, neither of the two datasets we describe in this paper offers definitive support for the environmental intolerance approach, with divergent findings between noise and olfactory data. Of relevance is a well-designed study by Lercher [50], which demonstrated that while those who repeated higher levels of noise annoyance more likely embraced coping strategies and complained about other traffic-related irritants (*i.e*., global sensitivities), those reporting greater sensitivity to noise engaged less coping strategies and complained less about traffic-related irritants, but reported increased sleep disturbance and health issues. 

The clinical importance of noise sensitivity has arguably yet to be realized [51], and while some argue that noise sensitivity is a marker of susceptibility to health problems in general [52], or that the relationship between noise sensitivity and health outcomes largely reflect negative affectivity [37], such propositions are not always supported in the literature. For example, in a study on road noise, Welch *et al.* [35] demonstrate that noise sensitivity moderates the relationship between noise exposure and health outcomes, rather than merely marking a predisposition to health deficits [52]. The small variance-accounted-for statistics that typically accompany significant coefficients marking the relationship between noise sensitivity and sensitivity to other sensory dimensions (e.g., olfaction) suggest that the Environmental Intolerance approach cannot be considered a general principle, and indeed it may be that noise sensitivity can be explained by a multitude of mechanisms working independently or interactively. We consider two such mechanisms in this discussion: Negative affectivity and information processing, but must acknowledge the existence of other approaches as well. For example, Miedema and Vos [11,12] report the relationship between noise sensitivity and fear of harm from the noise source, while Paunović *et al.* [53] suggest that noise-sensitive individuals may associate road traffic noise with danger, thus providing an anxiety hypothesis of noise sensitivity.

The methodological limitations of the current study have been largely dealt with when interpreting the results of the two studies. One limitation common across both studies is that secondary analyses often suffer from loose operationalization, forced by the deployment of *post hoc* analyses that were not envisaged as part of the original study. However, our results do lend themselves to suggest future directions of study. Information processing approaches indicate that noise sensitivity may not always be a higher-order (*i.e.*, evaluative) phenomenon, as argued by the NA hypothesis. Rather, pre-attentive processes, such as those occurring in the thalamus (*i.e.*, sensory gating), and likely genetic in origin, may partly underlie this vulnerability. Belojevic et al., [10] bemoan the lack of attention to noise annoyance as a mediator between noise exposure and information processing. They describe a number of potential mechanisms in which annoyance could impair cognitive function, including distraction, masking of goal-relevant information, and increased cognitive load. Thus, future development of self-report noise sensitivity scales might include items that not only probe negative evaluation of noise and other sensory dimensions, but also the integrity of cognitive function when exposed to noise and other environmental stimuli.

## 7. Conclusions

Two secondary analyses were performed on affective ratings of sensory data, specifically noise annoyance data and affective ratings of odor pleasantness (Study One) and air quality (Study Two). The research hypotheses were motivated by explanations of noise sensitivity focusing on negative affect. Findings from all analyses indicate that, in itself, negative affect is unlikely to be a general cause of noise sensitivity, and other mechanisms are worthy of examination. Specific challenges needed to be addressed in future research center on the theoretical and empirical disentanglement of negative affect, global sensitivities, and other potential mechanisms of noise sensitivity. This will likely entail refining analyses to specific subgroups, who may represent distinct etiologies in relation to their sensitivity to noise. 
